# OpenTrials: towards a collaborative open database of all available information on all clinical trials

**DOI:** 10.1186/s13063-016-1290-8

**Published:** 2016-04-08

**Authors:** Ben Goldacre, Jonathan Gray

**Affiliations:** Centre for Evidence Based Medicine, Department of Primary Care Health Sciences, University of Oxford, Oxford, OX2 6GG UK; Department of Non-Communicable Disease Epidemiology, London School of Hygiene and Tropical Medicine, Keppel Street, London, WC1E 7HT UK; Policy and Research, Open Knowledge, St John’s Innovation Centre, Cowley Road, Cambridge, CB4 0WS UK

## Abstract

OpenTrials is a collaborative and open database for all available structured data and documents on all clinical trials, threaded together by individual trial. With a versatile and expandable data schema, it is initially designed to host and match the following documents and data for each trial: registry entries; links, abstracts, or texts of academic journal papers; portions of regulatory documents describing individual trials; structured data on methods and results extracted by systematic reviewers or other researchers; clinical study reports; and additional documents such as blank consent forms, blank case report forms, and protocols. The intention is to create an open, freely re-usable index of all such information and to increase discoverability, facilitate research, identify inconsistent data, enable audits on the availability and completeness of this information, support advocacy for better data and drive up standards around open data in evidence-based medicine. The project has phase I funding. This will allow us to create a practical data schema and populate the database initially through web-scraping, basic record linkage techniques, crowd-sourced curation around selected drug areas, and import of existing sources of structured and documents. It will also allow us to create user-friendly web interfaces onto the data and conduct user engagement workshops to optimise the database and interface designs. Where other projects have set out to manually and perfectly curate a narrow range of information on a smaller number of trials, we aim to use a broader range of techniques and attempt to match a very large quantity of information on all trials. We are currently seeking feedback and additional sources of structured data.

## Background

Trials are used to inform decision making, but there are several ongoing problems with information management on clinical trials, including publication bias, selective outcome reporting, lack of information on methodological flaws, and duplication of effort for search and extraction of data, which have a negative impact on patient care. Randomised trials are used to detect differences between treatments because they are less vulnerable to confounding, and because biases can be minimised within the trial itself. The broader structural problems external to each individual trial result in additional biases, which can exaggerate or attenuate the apparent benefits of treatments.

To take the example of publication bias, the results of trials are commonly and legally withheld from doctors, researchers and patients, more so when they have unwelcome results [1, 2], and there are no clear data on how much is missing for each treatment, sponsor, research site, or investigator [3], which undermines efforts at audit and accountability. Information that is publicly available in strict legal terms can still be difficult to identify and access if, for example, it is contained in a poorly indexed regulatory document or a results portal that is not commonly accessed [4, 5]. In addition to this, different reports on the same trial can often describe inconsistent results because of, for example, diverse analytic approaches to the same data in different reports or undisclosed primary outcome switching and other forms of misreporting [4, 6]. There is also considerable inefficiency and duplication of effort around extracting structured data from trial reports to conduct systematic reviews, for example, and around indexing these data to make it more discoverable and more used. Lastly, although large collections of structured “open data” on clinical trials would be valuable for research and clinical activity, including linkage to datasets other than those on trials, there is little available and it can be hard to search or access.

In 1999, Altman and Chalmers described a concept of “threaded publications” [7], whereby all publications related to a trial could be matched together: the published protocol, the results paper, secondary commentaries, and so forth. This suggestion has been taken up by the Linked Reports of Clinical Trials project, a collaboration of academic publishers which was launched in 2011 with the aim of using the existing CrossMark system for storing metadata on academic publications as a place where publishers can store a unique identifier (ID) on each trial to create a thread of published academic journal articles [8].

We have obtained funding for phase I of a project that expands this vision, going further than linking all academic papers on each trial: an open database of all structured data and documents on all clinical trials, cross-referenced and indexed by trial. The intention is to create a freely re-usable index of all such information to increase discoverability, facilitate audit on accessibility of information, increase demand for structured data, facilitate annotation, facilitate research, drive up standards around open data in evidence-based medicine, and help address inefficiencies and unnecessary duplication in search, research, and data extraction. Presenting such information coherently will also make different sources more readily comparable and auditable. The project will be built as structured “open data”, a well-recognised concept in information policy work described as “data that can be freely used, modified, and shared by anyone for any purpose” [9].

This article describes our specific plans, the types of documents and data we will be including, our methods for populating the database, and our proposed presentations of the data to various different types of users. We do not have funding to manually populate the entire database for all data and documents on all trials, and such a task would likely be unmanageably large in any case. In the first phase, we aim to create an empty database with a sensible data schema, or structure, and then populate this through a combination of donations of existing sets of data on clinical trials, scraping and then matching existing data on clinical trials, with the option for users of the site to upload missing documents or links, and manual curation for a subset of trials. We will also create user-friendly windows onto this data. Our project start date was April 2015; our first user engagement workshop was in April 2015; and, after consultation on features and design, our first major coding phase will start in September 2015. We are keen to hear from anyone with suggestions, feature requests, or criticisms, as well as from anybody able to donate structured data on clinical trials, as described below.

### Data schema

**Fig. 1:**
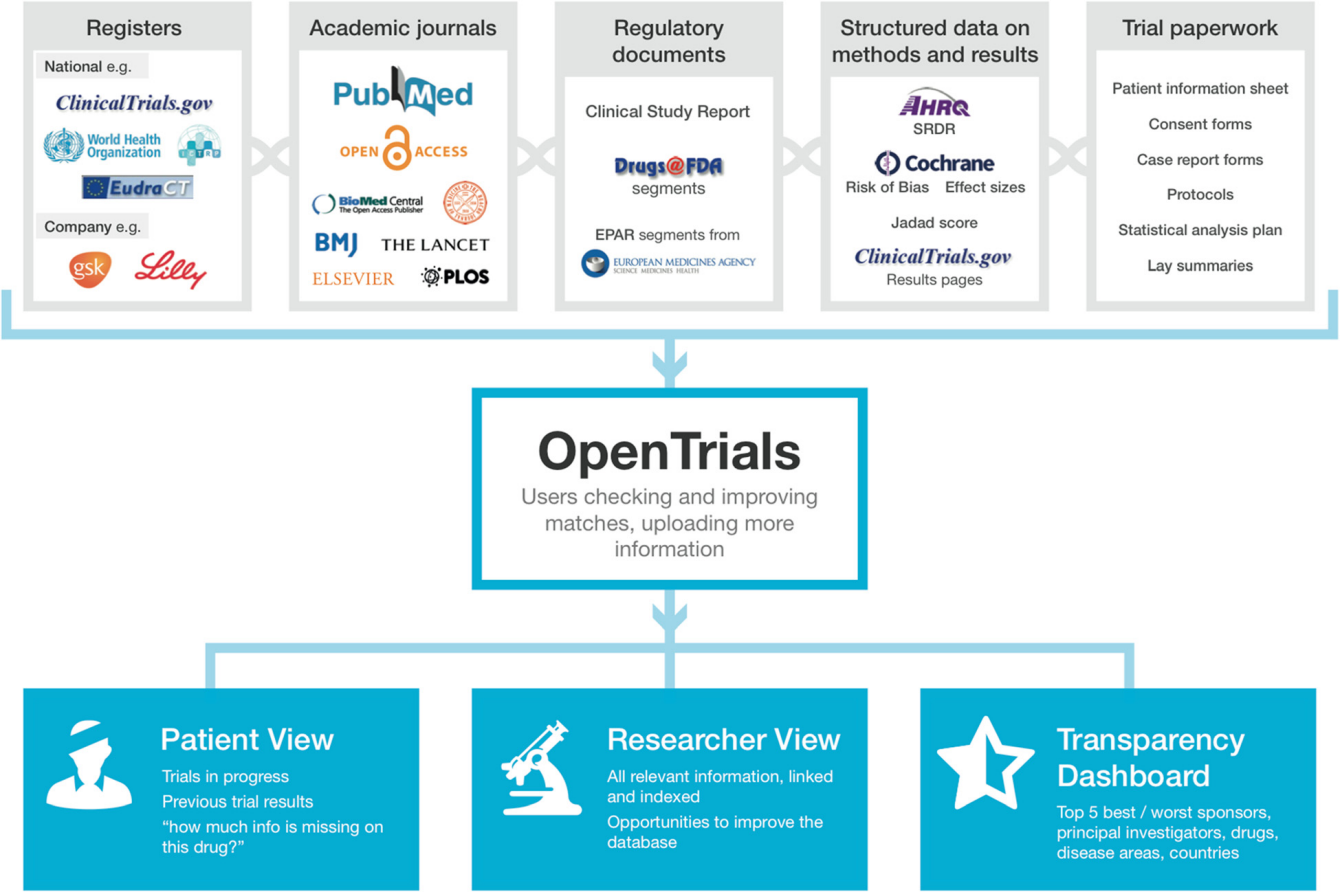
Overview of OpenTrials data schema and information flow

A description of the main classes of documents and data included is presented below and in Fig. 1. In overview, where possible, we will be collecting and matching registry entries; links, abstracts, or texts of academic journal papers; portions of regulatory documents describing trials; structured data extracted by systematic reviewers or other researchers; clinical study reports; additional documents such as blank consent forms; and protocols.

### Types of documents and data included

*Registers* are a valuable source of structured data on ongoing and completed trials. There are two main categories of register: industry registers, containing information on some or all trials conducted by one company, and national registers, containing information on some or all trials conducted in one territory or covered by one regulator. National registers generally consist of structured data on 20 standard data fields set out by the World Health Organisation (WHO) [10]; industry and specialty registers are more variable [11]. The WHO International Clinical Trials Registry Platform is a “registry of registers” combining the contents of a large number of registers in one place [12]. The simple act of aggregating, deduplicating, and then comparing registers can in itself be valuable. For example, in preliminary coding and matching work, we have found that trials listed in one register as “completed” may be listed as “ongoing” in another; thus, anyone looking only in the register where the trial was “ongoing” would not have known that results were, in fact, overdue. Similarly, where the text field for primary outcome has been changed during a trial, this can be identified in serial data on one registry and flagged up on the page for that trial. Registers presenting structured data have consistent and clearly denoted fields containing information on features such as the number of participants, the interventions (ideally using standard dictionaries and data schemas for consistency with other structured data), inclusion and exclusion criteria, primary and secondary outcomes, location of trial sites, and so forth. This information is ready to be extracted, processed, or presented. As a very simple example, after extracting this information, one can calculate the total number of trial participants on an intervention globally, restrict a search to include only large trials, or facilitate search of ongoing trials within 50 miles of a location, on a specific condition, where data quality permits [13].

*Academic journals* are one source of information on clinical trials, in the form of semi-structured free text, although they have increasingly been found to be flawed vehicles for such data. For example, they are less complete than clinical study reports [14], inconsistent with mandated structured data on registers [15], and permissive on undisclosed switching of primary outcomes [6] and other forms of misreporting [16]. Journal articles on trials include other document types, such as commentaries and protocols. Academic journal articles reporting trial results can be matched against registry entries through various imperfect techniques, such as searching for trial ID numbers in metadata on PubMed (for very recent publications only) while applying standard search filters for trials, or using record linkage techniques on other features such as intervention or population.

*Regulatory documents* are an important and often neglected source of information on trials. Clinical study reports are extremely lengthy documents produced for industry-sponsored trials. They have a closely defined structure, which academic researchers have recently begun to access more frequently [14, 17]. At the other end of the spectrum for length, there will often be free text descriptions of the methods and results of clinical trials mixed in with other information in bundles of regulatory documents released by the U.S. Food and Drug Administration and indexed on the Drugs@FDA website [18] or as part of the European public assessment report published by the European Medicines Agency for approved uses of approved drugs [19]. These documents are generally neglected by clinicians and researchers [5], poorly indexed, and hard to access and navigate. For example, the description of one trial may be buried in a few paragraphs in the middle of a long and poorly structured file, containing multiple documents, each covering multiple different issues around the approval of a product [4].

S*tructured data* on the results of clinical trials is available from two main sources: registers that accept results reporting, such as ClinicalTrials.gov and ISRCTN (International Standard Randomised Controlled Trial Number), and structured data that has been manually extracted from free text reports on trials by researchers conducting systematic reviews or other research. This can include structured data on the characteristics of the trial (such as number of participants or a description of the interventions using standard dictionaries) or the results of a trial (to populate fields in meta-analysis software), as well as data on the conduct of a trial or its methodological shortcomings; for example, many trials have had their risk of bias graded on various aspects of trial design using standard tools such as the Cochrane Risk of Bias Assessment Tool. There is also a Systematic Review Data Repository (SRDR) archiving structured data that has been extracted manually in the course of producing systematic reviews. SRDR is managed by the Agency for Healthcare Research and Quality (AHRQ), which has already begun to pool such data [20].

*Trial paperwork* includes protocols, lay summaries, and statistical analysis plans, as well as documents often currently regarded as “internal”, such as blank case report forms, blank consent forms, ethical approval documents, and patient information sheets. These are generally poorly accessible and rarely indexed, but they can contain salient information. For example, it was only by examination of case report forms that the team conducting the Cochrane review on oseltamivir and complications of influenza were able to establish that the diagnostic criterion for pneumonia was “patient self-report” rather than more conventional methods such as chest x-ray, sputum, and/or medical examination [21]. As another example, when presented with a trial in which the control group received a treatment which seems to be lower than the usual standard of care, a researcher or other interested party may wish to see the consent form to establish whether the benefits and risks of participation were clearly explained to patients. Lastly, ethics committee or institutional review board paperwork may contain information on how any potential risks were discussed or mitigated or may act as an additional source of information to identify undisclosed switching of primary and secondary endpoints. By placing all of this information side by side, identifying such inconsistencies becomes more straightforward and therefore may reasonably be expected to become more commonplace.

### Populating the database

Manually populating the database for all documents and data on all trials would be desirable, but it would be a major information curation project requiring very significant financial support. We initially aim to populate the database in sections, with breadth and depth in different areas, through a range of approaches, including web-scraping, basic record linkage techniques, curated crowd-sourcing, and imports or donations of existing structured and linked data.

*Importing publicly accessible structured data* is a straightforward way to initially seed a database of information on clinical trials. For example, the entire database of structured data on ClinicalTrials.gov can be downloaded and re-used. This database contains structured data on features such as title, number of participants, inclusion and exclusion criteria, interventions, outcomes, and so forth [22]. There are several other sources of structured data that can be downloaded and re-used under standard Creative Commons licenses for non-commercial re-use with attribution, such as the SRDR archive hosted by AHRQ [20]. Where structured data or documents on trials are publicly accessible but not available for download as a single coherent dataset, *web-scraping* can be used. This is a well-established technique whereby large quantities of structured data can be downloaded from websites automatically using scripts to visit large numbers of web pages sequentially and to download data from tables in pages.

Once data about trials are obtained, the issue then becomes matching data on each individual trial from the various different sources, such as matching a ClinicalTrials.gov registry entry against a row of manually extracted data on results that has been downloaded from SRDR. This is a *record linkage* issue, and there is a long and established literature and code base on the subject in other domains, such as patient records. Where two records share a common ID, such as a clinical trial ID number, they can simply be merged. If there is no common unique identifier, then standard probabilistic record linkage techniques can be used on various features of the trial.

An extension of this technique can be used for targeted web-scraping. For example, all academic papers in PubMed published since 2007 that refer to a registered trial should contain the trial registry ID in the XML data of the PubMed entry (although compliance with this feature was poor initially and has improved over time). International Committee of Medical Journal Editors guidelines have stated since 2005 that all trial results reported in journals should include the trial registry ID in the abstract. Therefore, we can automate a search of PubMed to identify academic publications with a given trial ID and import or generate metadata on these documents to our thread for that trial, including the type of publication (such as protocol, results, or commentary), year of publication, author names, and journal title. Linkage of PubMed and ClinicalTrials.gov has already been successfully conducted elsewhere [23], and the use of record linkage and targeted scraping techniques can be extended to other data sources.

We will also facilitate *curated and targeted crowdsourcing*. On the main page for a trial, in our current design, there is a list of documents and data we would like to have for each trial and an icon denoting whether it is present. If it is not present, there is an “upload arrow”. As an illustration, where we have a trial thread that contains a registry entry and an academic publication on results, but nothing more, then visitors can click to upload something such as a file containing structured data on results, a link to a clinical study report that they have located online, or a copy of a blank consent form. Each upload requires metadata, checking, and credit where necessary, with the option for users to flag where things have been incorrectly associated with a trial. While participatory data curation brings challenges, there is a large and growing knowledge base on this approach, both from Open Knowledge directly [24] and more broadly in the open data community.

We have also initiated collaborations around *donations of structured data*. There are many large datasets around the world where some form of record linkage has been done manually, or where structured data has been extracted from free text, to conduct a single piece of research. For example, large samples of registry entries on completed trials have been matched to academic publications and other sources of results on a specific search date to create cohorts to investigate publication bias. We have already arranged donations from researchers of three datasets of varying sizes covering varying types of data in various fields. Where disparate records pertaining to a single trial have been matched manually in this fashion, that matched data can be used in turn to validate automated record linkage techniques. It is important that the contribution and investment by those who have created such datasets be recognised and rewarded [25] while also ensuring that maximum patient benefit is derived from their work, minimising duplication of effort. By maintaining metadata on provenance, we are able to proactively give credit for all donated, imported, and externally linked data, wherever data are presented or downloadable. We are working with initial data donors on ways to do this most effectively, such as by giving credit to sources on the page for a specific trial and automatically generating a bespoke list of required acknowledgements and references for secondary users when a batch of data is downloaded and re-used. Notably, all researchers who have so far shared data in the preliminary stage of OpenTrials have expressed enthusiasm for greater public benefit from the effort which went into creating their dataset, especially as in some cases the only previous output from the creation of a large threaded dataset was a portion in a table in a published academic paper. One researcher group has expressed concern about their data being downloadable for re-use by other researchers before they have extracted adequate value from it, which is a common and legitimate concern in sharing raw data on all academic work [25]; researchers are sharing, but with a time delay.

Lastly, we are keen to populate the database manually, as perfectly as possible, and for a small number of trials to demonstrate the value of such a resource. There are only limited resources for this in phase I funding, but we will be guided in our choice of area by sources of funding and collaborations.

We currently intend to populate the database solely for randomised trials in humans; however, because this is principally a technical service rather than a manually curated library, any increase in volume is unlikely to materially affect the feasibility of the project. We are therefore open to expanding this remit to include other types of trials. For the same reason, there is no time limit on the era of trials that can be added or on the geographical territory covered.

### Presenting the data

We have developed prototype presentations of the data for different audiences and are currently running a series of user engagement workshops to improve these. Initial views are focused on search; researchers’ needs for individual trials; patients’ needs for individual trials; and overviews of performance metrics, which include transparency metrics on how much information is available for various classes of trial by sponsor, site, and so forth.

The webpage for researchers on a single trial is presented in Fig. 1. Across the top is the title and some basic information about the trial, extracted from a registry entry or a hierarchy of alternative sources. Below is a series of icons showing the headline documents and bundles of structured data that we would like to have on all trials. These icons are green if the relevant data or documents are present, and visitors can click through to view them; they are amber if the documents have been submitted or matched but not validated; and they are red if they are outstanding. Upload arrows are available for all missing documents so that they can be uploaded, as documents or links, by anyone who wishes to contribute.

Below that, we have various different proposed methods of presenting structured data. For example, where a trial’s risk of bias has been manually assessed somewhere and that data has been imported, we can display this in free text or icons to the visitor, showing them at a glance whether the trial has significant methodological shortcomings and what those shortcomings were. We can also predict whether individual patient data (IPD) should be available for the trial on request and guide the visitor to the relevant portal (of which there are currently at least 12), using simple algorithms running on the structured data. For example, if a trial is conducted after 2007, for a currently marketed product, and sponsored by GlaxoSmithKline, the IPD should be available on request through ClinicalStudyDataRequest.com, and contextual explanatory notes for this service are also provided. This may help to increase the use of such data, which is only requested infrequently at present.

**Fig. 2:**
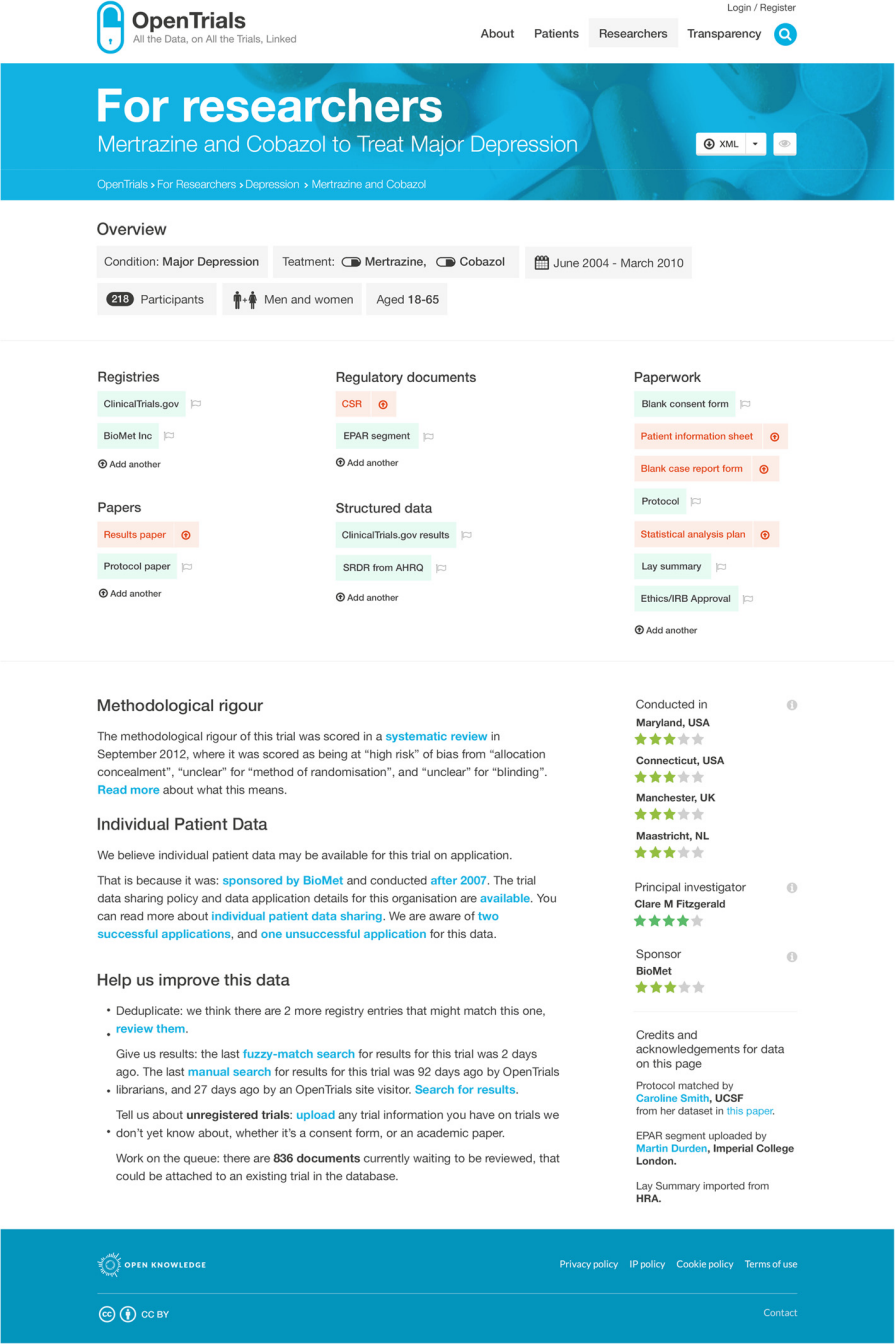
OpenTrials researcher view on a single trial (using mock data for feedback on proposed design only)

The presentation for patients (Fig. 2) is limited by the quality of the data currently available for this audience, but it has significant potential with greater user engagement. For example, we can present search options for ongoing trials for a given condition or a given drug, covering a given geographical area, filtered if necessary for an individual’s eligibility by comparing their entered demographic information against structured data on the inclusion and exclusion criteria of each trial, where data quality permits. Previous efforts to do this have been hindered by the variably poor quality of information on registries for non-specialist users. Here there are many opportunities. The first is from record linkage. For example, all trials must pass through an ethics committee, and all ethics committees require a lay summary. Where we can match the lay summary from ethics committee paperwork, we can present it on the patient-facing page. The second opportunity comes from using the option of crowd-sourcing and annotation, as we can also permit others to upload their own lay summaries. To this end, we have begun negotiating with science communication course leaders to work with them on using this as an exercise for their students, and are also keen that methodological shortcomings in ongoing and completed trials be communicated clearly to patients, with a view to developing a good trials guide. Here, as with other additional features to the core service, our efforts will be driven by opportunities for collaboration.

**Fig. 3:**
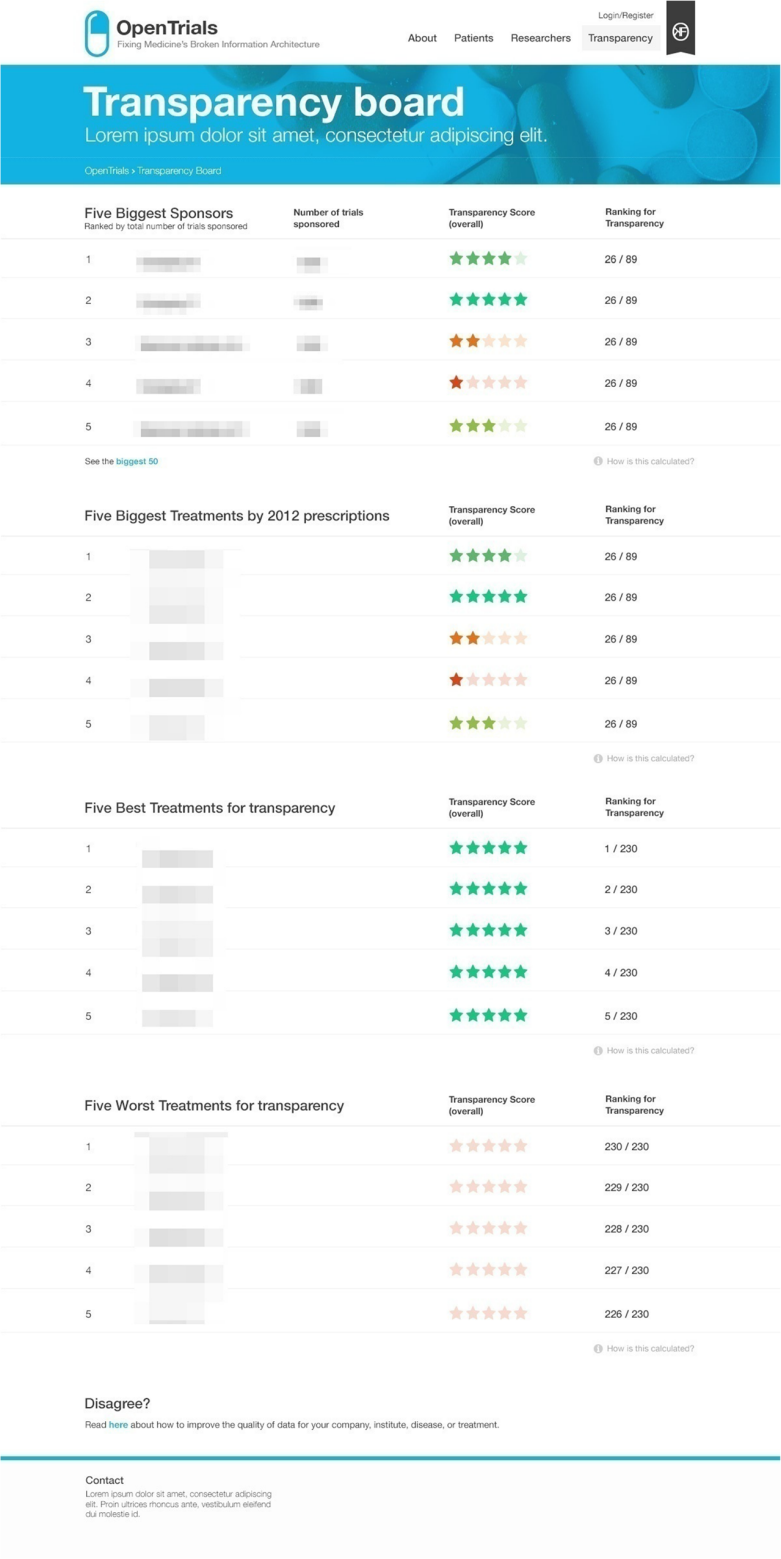
Transparency leader board (using mock data for feedback on proposed design only)

The overview of performance metrics (Fig. 3) demonstrates the value of having a large quantity of structured data in one place. For example, we can trivially produce dashboards reporting numbers of ongoing and completed trials but also, for areas or drugs where the data is reasonably complete, present metrics on transparency, such as showing how much information is currently missing for a given drug, sponsor, institution, investigator, and so forth. Such leader boards may be instrumental in driving up standards on transparency [3].

### Some use cases

We envisage a wide range of users exploring a wide range of questions and are keen to hear from potential users with specific feature requests early in the development process to ensure that we can meet their needs. Some examples of use cases are presented here for illustration.

A *researcher* or *clinician* may wish to find out more about a range of trials on a drug, searching by various different features such as inclusion and exclusion criteria to match a specific population. For each individual trial, where it has already been manually graded for methodological rigour, the researcher is provided with this information immediately. Where the trial has been included in a systematic review, a link to the review is prominently displayed. If IPD is available on request, the researcher can see this immediately. Where the results on a trial have been reported in multiple different places, a researcher can rapidly review these side by side; if there are discrepancies, these may be informative. For example, there may be a more conservative analytic strategy used in the regulatory filing than in the academic paper, resulting in conflicting effect sizes or participant counts; the primary outcomes may be switched or conflict between different presentations of the results; or the names of authors and investigators may vary widely between registration and various presentations of results. Each of these elements may raise concerns for further investigation.

A *patient* interested in participating in a trial may visit the site looking for trials in progress, in their local area, and on their medical condition. A science communication or clinical trials master of science *student* may visit the site to identify a trial that is lacking a lay summary or expert review and then write one as a learning experience and for the benefit of the wider community. An *expert patient* or *policy officer* working for a *patient group* may research a range of trials on the medicines taken by patients with their condition and find that there are many trials completed for which apparently no results have been posted. They can conduct a brief search for missing results and post any results they are able to find. Should this search yield no results, or if a professional search has already been conducted on a recent date and confirmed no results, then the patient or patient group can contact the sponsor, principal investigator (PI), or company, explaining that they represent patients using this treatment and asking them to make the results of the trial publicly accessible.

A *healthcare worker in a developing country* setting may be told of an ongoing trial by a patient and be shown a consent form or patient information sheet. Such a person can upload a copy of that document, and it will be entered into the queue of unresolved submitted documents. Here it can be seen and checked whether it matches an ongoing registered trial. If it appears to be for an unregistered trial, a new holding ID can be assigned and a new thread commenced for that trial. In this way, the OpenTrials database can facilitate field surveillance for ongoing unregistered and therefore poorly regulated or unethical research.

*Trial sponsors* or *university research staff* may visit the site to ensure that all their trials have results publicly available, that all other data are available, and that registry entries are not conflicting. A *journalist* or *policy officer* interested in publication bias may visit the site and explore the treatments, PIs, sites, or sponsors with the highest rates of apparently unreported results on completed trials.

A *systematic reviewer* seeking to conduct a rapid review may visit the site to search for trials and to aggregate existing extracted structured data from the site to avoid duplication of effort, before generating structured data themselves on uncoded trials and then sharing this data in turn. A researcher working on *automating systematic reviews* may use manually extracted structured data on the site, matched to free text documents, to calibrate their automated data extraction algorithms and request bespoke fields to share their extracted data back to a hidden part of the site for shared comparisons among automated review researchers.

### Technical issues with data curation from multiple sources

Hosting a broad range of data and documents presents some challenges around curation, especially because different sources of structured data will use different formats and different dictionaries. Although we will exploit available mapping between different data schemas and dictionaries, we do not expect to necessarily make all sources of all structured data on all trials commensurable and presentable side by side. For example, intervention may be described in free text or as structured data using various different dictionaries, and even sample size may be labelled in different ways in different available datasets, not all of which can necessarily be parsed and merged. For simplicity, we are imposing a series of broad categories as our top-level data schema, following the list given above. This is best thought of as a thread of documents on a given trial, where a “document” means either an actual physical document (such as a consent form or a trial report) or a bundle of structured data for a trial (such as the structured results page from a ClinicalTrials.gov entry in XML format or a row of extracted data with accompanying variable names for a systematic review). This is for ease of managing multiple data sources, providing multiple bundles of structured data about each trial in multiple formats, each of which may be commonly or rarely used.

Parsers for such bundles of structured data, and mechanisms to present it in a user-friendly fashion, will be built according to need as expressed in our user groups. For example, we will parse ClinicalTrials.gov results pages in some detail and extract data on important features, such as sample size or primary and secondary outcomes, to present these on the page, because these data are consistently structured, well-curated, and available for a large number of trials. For more uncommon formats of structured data provided by systematic reviewers, we will extract some data or give options to present it on the page attractively (for example, listing “variable name” and “value”), but we will not present it on the main page for that trial. For more obscure structured data, such as the extracted data on a relational database used by a team of systematic reviewers internally (many of which may never have been included in a systematic review or a registry), we will extract some data from some fields and present these cleanly on the page but leave the rest available for download. Where anyone can provide us with a key to accompany their data schema, explaining what each variable name denotes, we will present that alongside their data. Overall, this approach represents a balance between what is achievable and perfect data curation, reflecting the fact that many users of complex structured data will be capable of using that structured data in its more raw forms.

Inconsistent structured data presents a further challenge, but also an opportunity. For example, “number of participants” may be slightly different in different data sources. This presents a challenge in terms of record linkage validating a match between data sources to ensure that both records do pertain to the same trial. It also presents a challenge in terms of data presentation, as a choice must be made regarding which to present in a user-friendly front page for a trial. This is an example of the issues covered in our user engagement workshops. However, it also presents an opportunity to identify and flag inconsistencies in data on the same feature of the same trial in different places, to facilitate research on the reasons for this, and to establish whether such inconsistencies have resulted in bias.

By comparison, indexing and threading free text documents present far fewer challenges. For each uploaded document, we expect to have some metadata covering provenance, date of upload, type, any available structured data from the source (subject to the issues above), and some optional additional extracted data.

### Open data in medicine

Open data is a widely recognised concept outside medicine, but to date there has been relatively little activity around open data in healthcare, and in particular almost none on clinical trials. The concept of “open data” arose in the open source software movement and in public sector information policy work. It now refers to a rapidly growing set of ideals, norms, and practises for publishing information from government, academia, civil society, and the private sector. Open data principles and standards stipulate how information should be disclosed: in machine-readable formats, for example, and with open licenses that remove restrictions on re-use [9]. The removal of legal and technical restrictions on re-use is intended to facilitate new forms of collaboration, innovation and re-use of data, such as through analysis, new applications and services, or collaborative databases and data “ecosystems” which combine and curate data from multiple sources.

Existing notable examples of open data include the OpenStreetMap project, a collaborative open data project to create a free map of the world, integrating geospatial data from many different sources, including the public sector, private sector, researchers, individuals, and civil society organisations. To date, this project has over 2 million registered and contributing users, with their data widely used as an alternative to proprietary geospatial information providers [26]. The Wikidata project, a sister project to Wikipedia, curates statistical data from a variety of different sources and currently has had over 230 million edits from 15,000 active users [27]. Both of these projects have been relatively successful in aligning the activities of different users to facilitate the collaborative development of a shared resource which can be re-used and developed in a wide variety of different contexts. The integration of these projects into different applications, services and workflows has also contributed in turn to their further development, population, and sustainability.

We hope that the OpenTrials project can become a similar collaborative open database project for medicine, and that it can help to catalyse a better data infrastructure for information about clinical trials. While many existing databases are limited to specific use cases (such as for compliance with regulation or for particular research communities), there is an opportunity to create a shared data infrastructure for medicine through a combination of flexible and extensible schemas and data structures, user interfaces catering to different users and use cases, proactively seeking collaboration with organisations and researchers who operate in this area, and being responsive to their needs. This will entail not just the technical work of collation, cleaning and presentation of data from multiple sources but also the social and political work of aligning the interests and activities of different organisations, researchers and users around collaborative activity. Elsewhere, Open Knowledge (the organisation leading the technical aspects of building the OpenTrials database) has used the phrase *participatory data infrastructures* to describe flexible information systems—with their various technical, legal, administrative and social components—that are responsive to the needs and interests of multiple different users and groups [28]. By being responsive, the data infrastructure can be extended to include fields and indicators which are not currently captured in existing information systems, which can make it more useful as a research resource, a tool for driving policy change and improvement in data quality, or for other as yet unforeseen purposes. In addition to this, the very act of requesting shares of bulk data can itself be a positive forward push.

As a minimum, we hope that OpenTrials and related projects will contribute to advancing norms and practices around access to data and documents in medicine, including the expectation that such information will be shared as structured open data that can be more readily matched, analysed and collaboratively improved.

### Intellectual property and privacy

There are various intellectual property (IP) issues presented by such a database, such as regarding third-party IP in articles, documentary materials or datasets. There are various approaches to managing these issues. For example, if a copy of a consent form is made available to us by a trial participant, then we believe there is a clear public interest in its being publicly accessible and available for download (with personal information redacted where needed). However, such forms can be lengthy written documents published without explicit permission to republish or re-use. While it seems unlikely that anyone would have a sincere commercial IP reason to withhold such documents from public access, it is possible to have other reasons to prefer that they be kept inaccessible or to have a blanket policy on restricting third-party use of all documents or a preference to host it on their own service; therefore, they may use IP law to prevent it from being either hosted or shared with doctors, researchers, and patients.

Here we believe the most sensible option is to pursue a simple three-stage policy: (1) link out to such documents, wherever possible, if they are publicly accessible in any form, but take a copy for archive in case the publicly accessible version disappears; (2) host the text if such documents are not accessible, assuming good faith and public interest, but provide a service for “take down” requests; and (3) treat each request for withdrawal on a case-by-case basis, seeking funding for legal expenses to defend public interest as and where this seems appropriate.

With respect to privacy, we propose to avoid hosting IPD to protect patient privacy. Instead, we will link to sources where IPD is available upon request and monitor the availability of these sources.

### Practical issues

The project has received phase I funding from the Laura and John Arnold Foundation, given to Open Knowledge and the Centre for Open Science, with BG as principal investigator. User engagement, database design, front-end design and coding will be carried out by Open Knowledge, and the back-end database is provided by the Centre for Open Science. We have a small steering committee meeting regularly for the daily running of the project and a larger advisory group with a wide range of users and stakeholders for intermittent guidance on build, strategic direction and sustainability. In terms of outcome measures, we have targets for the quantity of data imported and the number of active users, as well as policy impacts, such as raised expectations of access to documents and around structured open data on clinical trials.

Our objective for phase I is to create a functioning database with a practical schema; populate it through scraping, record linkage, data donations, crowd-sourcing, and a small amount of pilot curation; and create user-friendly web interfaces onto the data. We believe that this will provide a clear working demonstration of the value of a matched and indexed database of all structured data and documents on all clinical trials, and that it will enable us to work towards obtaining further funding to populate the database—the key financial challenge—and develop new features to meet demand from researchers, clinicians, policy makers, patients and other users. We are also considering alternative options for sustainability, such as offering a paid service whereby OpenTrials librarians can curate and enter data as perfectly as possible for a given set of trials in exchange for a fee, enabling research sites or sponsors to facilitate access to information on their trials and demonstrate compliance and transparency, although this raises potential conflicts of interest that would need to be managed. If, after producing a functioning service, it proves impossible to make the project financially sustainable, then we have a no-cost wind-down plan in place, sharing all code and data to appropriate platforms (e.g., GitHub and Figshare). Where further features and infrastructure have been developed using functions on the site, we will aim to reserve a fund to permit a static archive with functioning APIs so that any other projects dependent on OpenTrials features or data can continue to operate.

There are several clear shortcomings and challenges to the OpenTrials plan which we have attempted to mitigate within the confines of limited funding as described above. These challenges include limitations on financial and person-time resources that prevent us from creating a comprehensive, manually curated library of all information on all trials; the challenges around ensuring integrity of material submitted openly online; the challenges of maintaining information infrastructure over a term that exceeds stand-alone academic project grants; and the challenges around engaging a community to solicit wider sharing of documents and structured data. We are keen to hear feedback on additional strategies to meet these challenges.

## Conclusions

We are building an open free database and web service to identify, aggregate, store, match, index and share all available documents and data on all clinical trials. We are keen to receive feedback on the current methods, design, and data schema; feature requests; offers or suggestions of further data sources; and collaborations or methods to expand or improve the specification. Progress can be viewed at www.OpenTrials.net where the service will be hosted. 

## Abbreviations

AHRQ: Agency for Healthcare Research and Quality
ID: identifier
IP: intellectual property
IPD: individual patient data
ISRCTN: International Standard Randomised Controlled Trial Number
PI: principal investigator
SRDR: Systematic Review Data Repository
WHO: World Health Organisation
